# A Case of Post-polypectomy Syndrome Following Endoscopic Resection of a Rectal Polyp

**DOI:** 10.7759/cureus.88425

**Published:** 2025-07-21

**Authors:** Faheem Anjum, Ayesha Muneer, Muhammad Usman, Arslan Arshad

**Affiliations:** 1 Internal Medicine, Salford Royal NHS Foundation Trust, Salford, GBR; 2 Gastroenterology, Salford Royal NHS Foundation Trust, Salford, GBR; 3 Elderly Medicine, Salisbury District Hopsital, Salisbury, GBR; 4 Emergency Medicine, Royal Albert Edward Infirmary, Wigan, GBR

**Keywords:** acute abdomen, colonoscopy complications, colorectal polyp, conservative management, endoscopic complications, endoscopic mucosal resection, post-polypectomy syndrome, rectal polyp, thermal injury, transmural burn syndrome

## Abstract

Post-polypectomy syndrome (PPS) is a rare yet significant complication of endoscopic polypectomy that can clinically mimic intestinal perforation. This report presents the case of an 85-year-old female who developed PPS following an elective endoscopic mucosal resection (EMR) of a 3 cm mid-rectal polyp. Her past medical history included asthma and epilepsy. The EMR was performed in a piecemeal fashion using snare tip soft coagulation, and the defect was closed with eleven endoclips. The patient initially remained stable post-procedure but developed pyrexia, drowsiness, and a Glasgow Coma Scale (GCS) score of 3/15 later that evening. Laboratory results revealed severe metabolic acidosis, elevated lactate, markedly raised inflammatory markers, and significant leukocytosis. A contrast-enhanced CT scan of the abdomen and pelvis showed no evidence of perforation or obstruction but did reveal rectal wall edema, fat stranding, and a small-to-moderate volume of pelvic free fluid. These findings, in conjunction with her recent procedure and clinical deterioration, led to a diagnosis of PPS. She was managed conservatively with intravenous fluids and piperacillin-tazobactam; she made a full recovery and was discharged within 72 hours. This report underscores the importance of early recognition and differentiation of PPS from true bowel perforation. PPS is typically self-limiting and can be managed non-surgically with close monitoring. A thorough awareness of the risk factors-such as large polyp size, use of thermal coagulation, and rectal location-can assist in early diagnosis and prevent unnecessary surgical intervention. This report highlights the need for high clinical suspicion, timely imaging, and prompt supportive treatment in patients who deteriorate following endoscopic interventions.

## Introduction

Colorectal polyps have the potential to develop into colorectal cancer in up to 70-80% of cases and are typically removed via colonoscopic polypectomy [1]. Endoscopic techniques such as endoscopic submucosal dissection (ESD) offer a minimally invasive approach with low morbidity and mortality for early-stage colorectal cancer. However, complications such as bleeding and perforation can still occur. Post-polypectomy syndrome (PPS), also known as post-polypectomy coagulation syndrome or transmural burn syndrome, is a rare but recognized complication following colonoscopic polypectomy [2-3]. Patients with PPS may present with abdominal pain, peritoneal irritation, fever, and leukocytosis-symptoms that closely resemble those of intestinal perforation, although no perforation is typically evident on imaging studies.

The underlying pathophysiology of the condition involves thermal injury caused by high-frequency electrical current during polypectomy, leading to transmural burns and localized peritonitis [4]. This injury extends through the colonic wall layers, resulting in systemic inflammation and peritoneal irritation. While PPS is generally self-limiting, severe cases may be encountered, with approximately 1% requiring hospitalization [5].

## Case presentation

**Figure 1:**
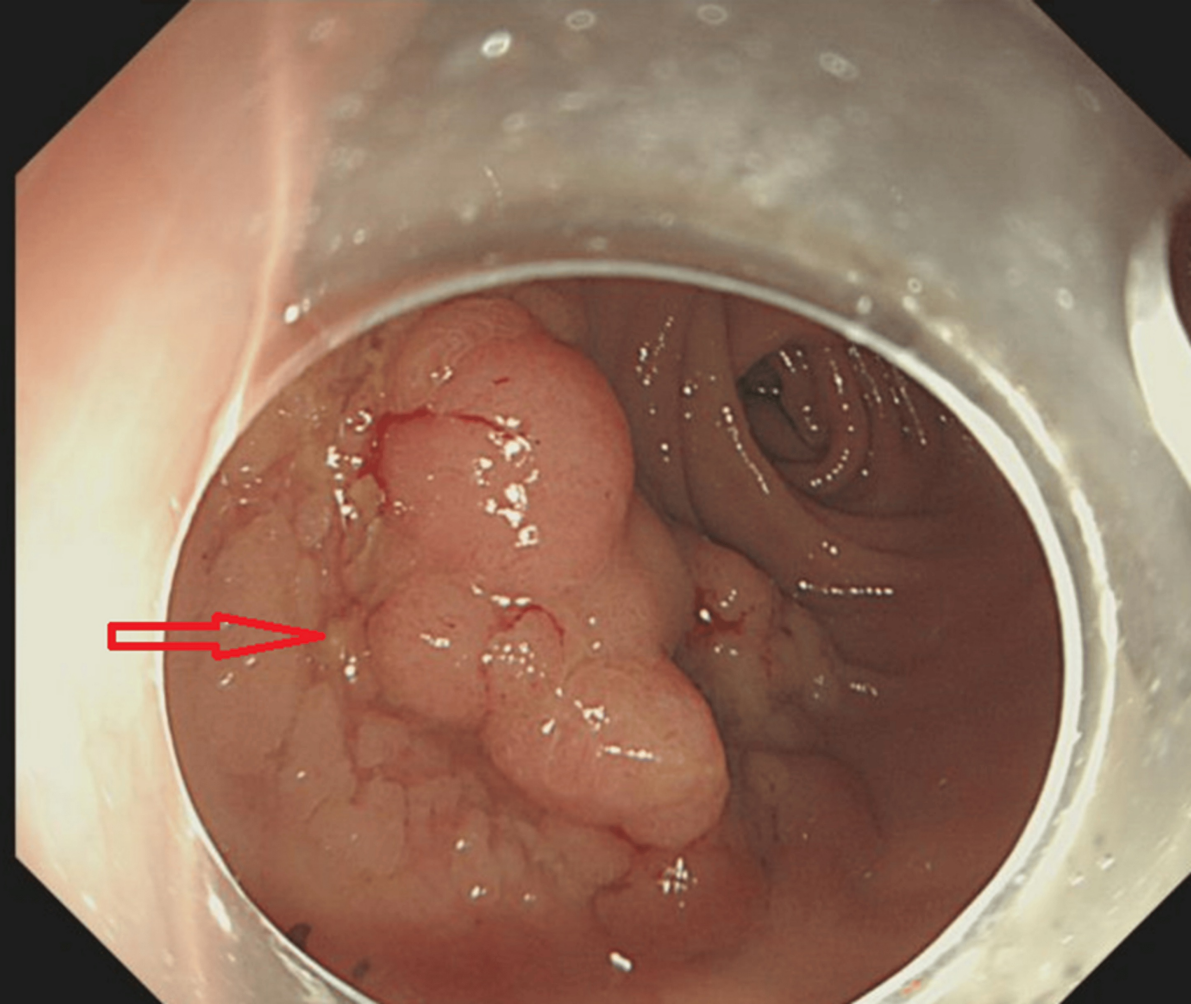
Endoscopic image showing a 3 cm mid-rectal polyp of villous morphology

**Figure 2:**
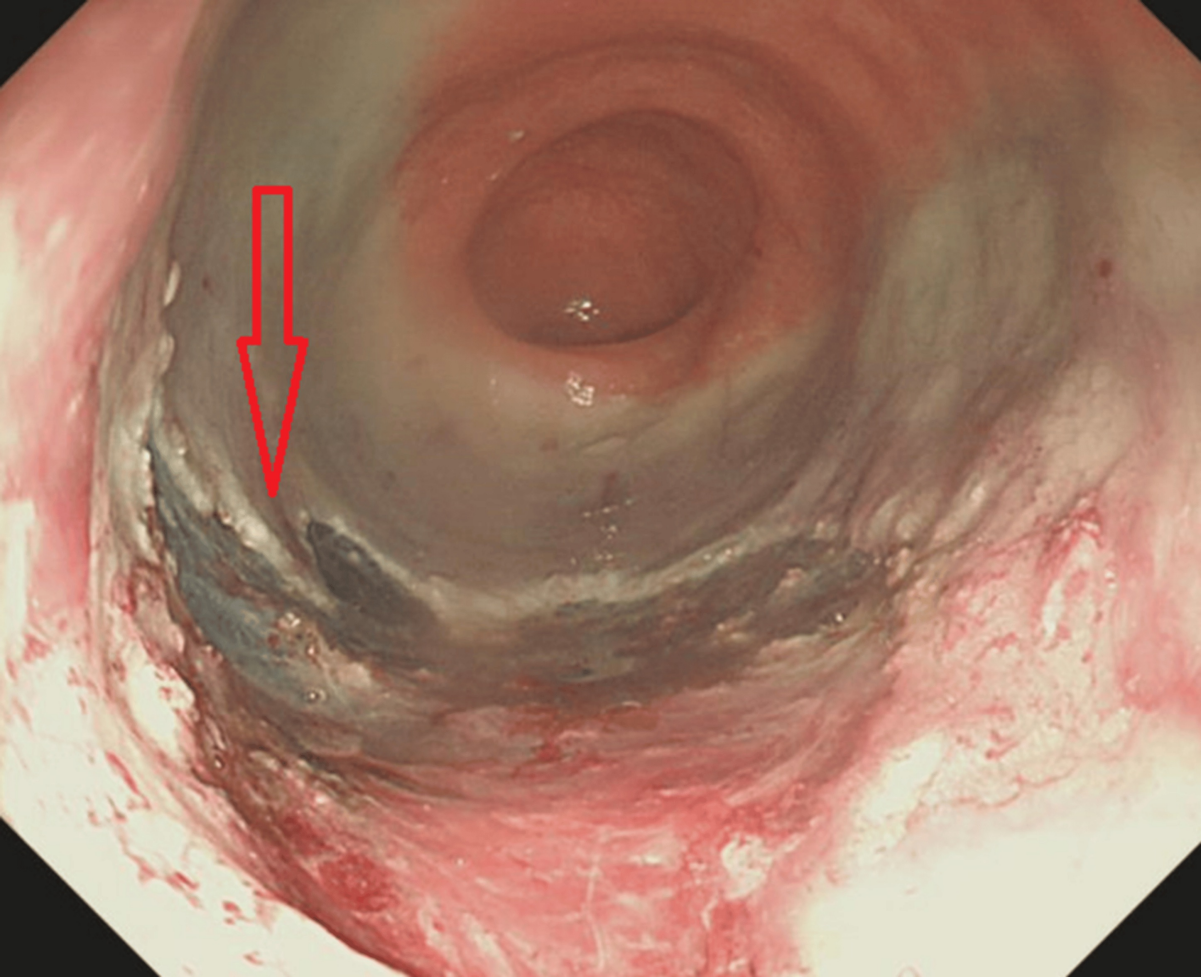
Post-resection endoscopic image showing EMR base EMR: endoscopic mucosal resection

**Figure 3:**
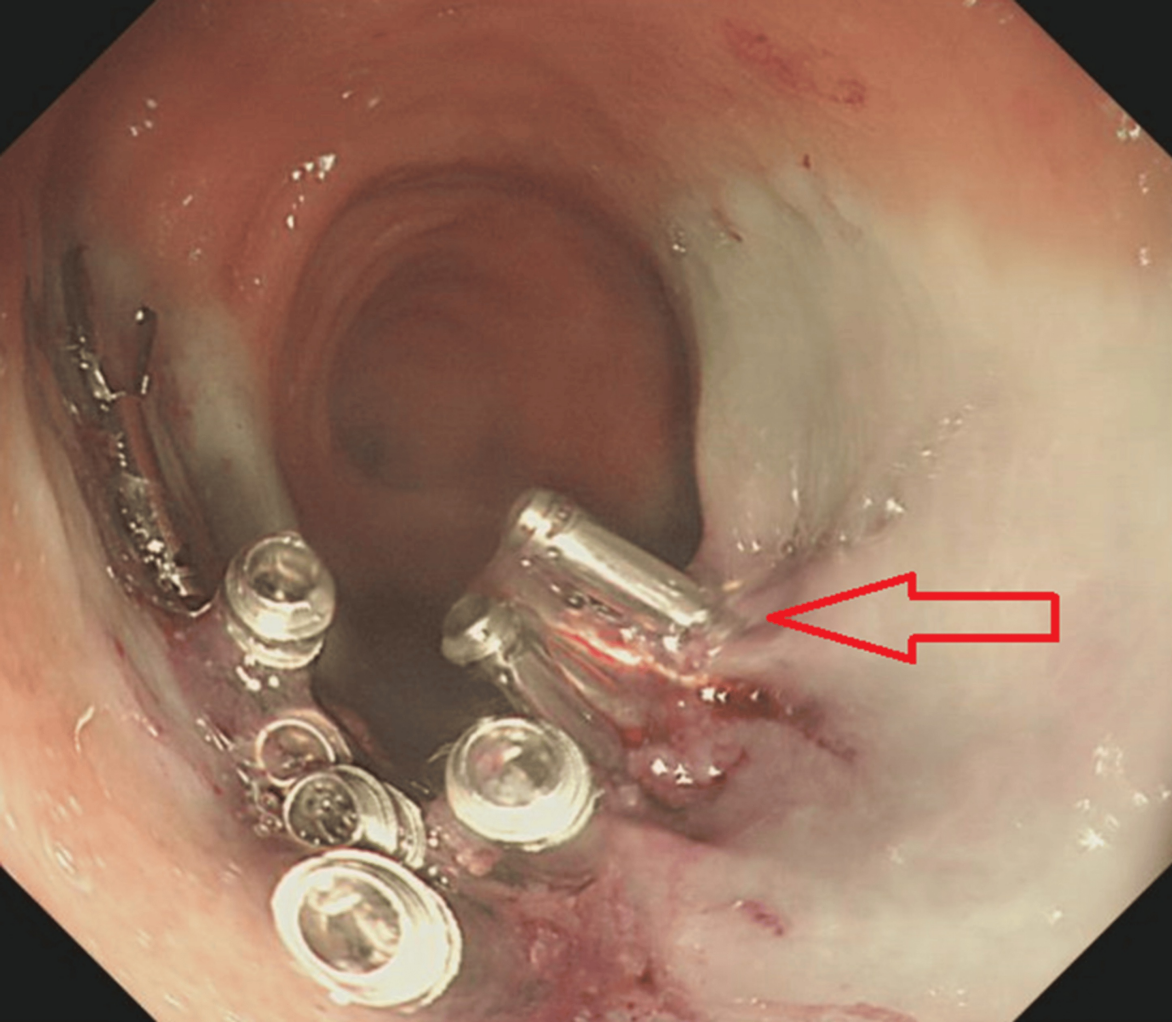
Image showing EMR base closed with clips EMR: endoscopic mucosal resection

An 85-year-old female who was known to have a 3 cm mid-rectal polyp was admitted for an elective polypectomy (Figure 1). Her medical history included asthma and epilepsy. Routine medications included levetiracetam, tiotropium bromide inhaler, fluticasone furoate/vilanterol combination inhaler, and mirabegron. She underwent a morning sigmoidoscopy during which a mid-rectal polyp was removed in a piecemeal fashion via endoscopic mucosal resection (EMR), followed by snare tip soft coagulation (Figure 2). The EMR base was closed using 11 clips (Figure 3). The patient remained stable immediately after the procedure and was transferred to the gastroenterology ward.

That evening, the patient developed pyrexia (temperature: 38.1 °C) and became drowsy and unresponsive within two hours. The hospital resuscitation team assessed her. Observations revealed a temperature of 38.5°C, a heart rate of 125 bpm, a blood pressure of 158/81 mmHg, and a respiratory rate of 24/min. There was no evidence of rectal bleeding. Abdominal examination revealed a soft, non-tender abdomen. Her Glasgow Coma Scale (GCS) score was 3/15. Blood investigations taken during clinical deterioration are shown in Table 1.

**Table 1:** Blood test results during clinical deterioration

Variables	Patient values	Reference range
PH	7.19	7.35-7.45
PO_2_	22.4 kPa	11.0-14.0 kPa
PCO_2_	4.1 kPa	4.6-6.0 kPa
HCO_3_	12 mmol/L	21-28 mmol/L
Serum lactate	16.7 mmol/L	<2 mmol/L
C-reactive protein	133 mg/L	<10 mg/L
White cell count	34.9 x 10^9^/L	(4.0-11.0)^9^/L

**Figure 4:**
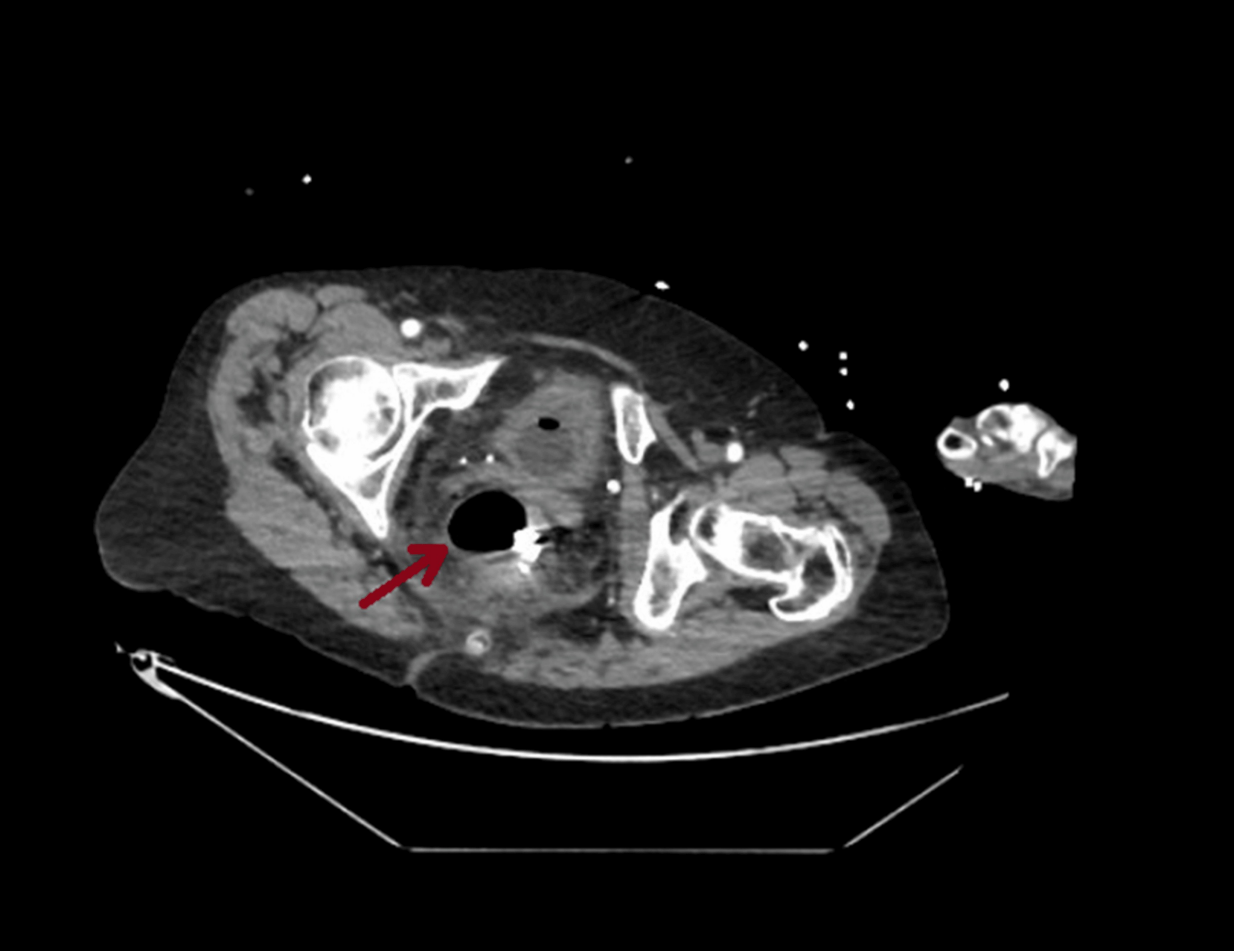
CT scan image showing edema and fat stranding around the rectum consistent with proctitis CT: computed tomography

A biphasic contrast-enhanced CT scan of the abdomen and pelvis revealed no bowel ischemia or obstruction. However, there was a small-to-moderate volume of pelvic free fluid with surrounding rectal edema and fat stranding, which was consistent with post-polypectomy site inflammation and proctitis (Figure 4).

A diagnosis of PPS was made based on symptoms, history of recent polypectomy, and the absence of intestinal perforation on the CT scan. Parenteral fluids and antibiotics (piperacillin-tazobactam) were initiated, leading to a full recovery. The patient was discharged 72 hours after admission.

## Discussion

PPS arises due to electrocoagulation injury to the muscularis propria and serosal layers, resulting in transmural burns. Symptoms typically manifest within 6-12 hours and may include abdominal pain, fever, vomiting, and signs mimicking sepsis. Laboratory findings often demonstrate elevated inflammatory markers. Risk factors for PPS include polyp size >20 mm, hypertension, right-sided lesions, and sessile morphology [6-7]. Although intestinal perforation and bleeding are well-known complications of polypectomy, PPS should be considered in any patient with acute abdominal symptoms following endoscopic intervention. CT imaging is the gold standard for differentiating PPS from perforation. PPS is typically characterized by mural thickening and fat stranding, without evidence of extraluminal air [8].

Management is largely conservative, consisting of bowel rest, intravenous fluids, and antibiotics. Patients with mild symptoms may be discharged earlier on oral antibiotics and a liquid diet. A small study by Yamasaki et al. suggested that line-assisted complete closure of mucosal defects post-ESD may reduce PPS incidence, although larger studies are needed to confirm these findings [9]. PPS, also known as transmural burn syndrome, is a rare complication with a reported prevalence ranging from 0.003% to 0.1% following colonoscopic polypectomy, particularly when using electrocautery for large sessile lesions [10].

## Conclusions

PPS, although rare, should be considered in the differential diagnosis of patients presenting with systemic inflammatory signs following colonoscopic polypectomy, especially in the absence of perforation on imaging. Early recognition is vital to avoid unnecessary surgical intervention. Conservative management with intravenous fluids, antibiotics, and close monitoring is often effective. This report highlights the importance of clinical vigilance and imaging in distinguishing PPS from more serious complications, thereby ensuring timely and appropriate treatment.
